# Soccer and Relative Age Effect: A Walk among Elite Players and Young Players

**DOI:** 10.3390/sports5010005

**Published:** 2017-01-11

**Authors:** Manuel Jacob Sierra-Díaz, Sixto González-Víllora, Juan Carlos Pastor-Vicedo, Jaime Serra-Olivares

**Affiliations:** 1 Physical Education Department, Teaching Training Faculty, University of Castilla-La Mancha, 16071 Cuenca, Spain; jacobsierradiaz@hotmail.com; 2 Physical Education Department, Teaching Training Faculty, University of Castilla-La Mancha, 45004 Toledo, Spain; juancarlos.pastor@uclm.es; 3 Department of Physical Education Pedagogy, Faculty of Education, Catholic University of Temuco, 02950 Rudecindo Ortega, Temuco, Chile; jserra@uct.cl

**Keywords:** relative age effect, football, sport talent, young and elite football players

## Abstract

Grouping people according to chronological age is popular in fields such as education and sport. Athletes who are born in the first months of the year usually have cognitive and physical development differences in contrast to those born in the last months of the same year. That is why competitive teams tend to select older players more often than youngsters. Age differences between athletes born in the same year as well as an over-representation of older players are known as the *Relative Age Effect*. This effect is extensively described in young and elite team sports such as basketball, volleyball or, ice-hockey, as well as in soccer. The purpose of this study is to examine the state-of-the-art of the Relative Age Effect in youth and elite soccer players. This review summarizes recent research articles on the Relative Age Effect related to competitive soccer from 2010 to 2016. The systematic literature search was conducted in four databases: SPORTDiscus, Medline, EBSCO host and Google Scholar. Although causes and final solutions have not been clearly achieved yet, it is necessary to continue investigating this phenomenon in order to provide a starting point for future research.

## 1. Introduction

Educators and trainers tend to group players by chronological age in order to ensure equal opportunities of success, which is achieved by establishing an “activity year” [1]. The teams are often organized into annual age-groups, for which 1 January is normally used as the cut-off date [2,3]. This concept is named ‘Relative Age’. It refers to the differences among the birth-months of the players during the same year. The consequences of this term are named ‘Relative Age Effect’ (RAE) [4,5]. In this regard, an over-representation of players born during the first two quarters of the year and an under-representation of players born during the last two quarters of the same year [3,6,7,8,9] is observed. RAE is especially relevant during the adolescence years, due to the fact that physical characteristics are related to an increased chronological age [10]. Then, athletes born earlier in the year have an advantage over those born later in the same year when they encounter a similar task or exercise [11]. However, RAE decreases after the adolescent period. This is explained by physical maturation being less determinant [10].

The Relative Age Effect has been identified in many strength-, endurance-, and technique-related sports, as well as in competitive forms [3] such as baseball [12], ice hockey [6], tennis [13] or soccer [11]. Although, this phenomenon has been observed in education as well [14], it is suggested that children who were born in January will have a whole year of maturation advantage compared to children born in December [15]. However, the RAE has not shown a serious impact during formal education or adulthood employment [16]. RAE has been investigated widely in male sports compared to female sports [17].

The first study about RAE and sport was supported by Barnsley and Thompson (1985) [18]. These authors analyzed the birthdates of hockey players in the United States. This study showed that players who were born during the first months of the year had advantages in comparison with those born during the last months, due to their participation in the competition. As a result, this could cause that the youngest players feel frustrated and finally leave their team. The first study about RAE and soccer was conducted by Barnsley, Thompson and Legault in 1992 [1]. They investigated under-20 and under-17 players who participated in the 1990 World Football Cup. Results indicated that those players born in the first months (quarters) of the year were over-represented, while those born in the last months of the same year were under-represented.

The Relative Age Effect in soccer has been identified among young and elite soccer players in several countries including Belgium, Denmark, England, France, Italy, the Netherlands, Sweden [11], Germany [19], Spain [20], Brazil [21], the United States [22] and Australia [23]. However, in sports where physical attributes such as size and body mass might be less likely to influence performance, for instance golf, the RAE has not been identified [24]. On the other hand, ‘inverse’ RAE (an over-representation of players in the final part of the year and an under-representation of players at the beginning of the same year) was found in non-physical sports activities including shooting sports [25].

It is possible to identify two types of factors which influence the degree of incidence of RAE in a specific sport. First, extrinsic factors, such as socioeconomic determinants, may increase competitiveness in access and opportunities during talent identification. Second, intrinsic factors, such as physical and psychological characteristics, may connect sport experience with talent [26]. For this reason, coaches are an important social agent of RAE biased selection of children with physical and maturation advantages [15]. That is also why researchers have wanted to know whether coaches are aware of RAE. The latest studies highlighted the importance of coach education in all fields of RAE, including the decision-making process during talent detection as well as various practical and managerial recommendations on coach organization [26,27,28,29].

For all the above, it is necessary to identify the presence of RAE in different contexts and at different ages. In this sense, the aim of this manuscript is to analyze the RAE in young football players (from 6 to 18 years old) and elite football players (more than 18 years old) as well as its possible solutions derived from the most recent studies. The main hypothesis is formulated as a significant increase of RAE on competitive soccer players from 2010 to 2016 around the world.

## 2. Methods

In this paper, results, conclusions and implications about RAE in young and elite soccer players during the last years were analyzed. In order to make a thorough review, the manuscript also follows some systematic review and meta-analysis recommendations [30].

### 2.1. Problem Formulation

This paper is a bibliographic review that tries to answer the questions below:

- Is RAE maintained across age categories?
- Is RAE present in female soccer players?
- Is RAE influenced by the player position on the pitch?
- Is RAE related to physical advantages of the older players in contrast to the young players born in the same year?
- Is RAE increasing during 2010 to 2016 around the world or is it influenced by the size of the country? Is there any solution which helps to reduce RAE?

Consequently, the main objective is to observe the presence of RAE in the latest studies as well as to assess which topics are related with this effect in order to explain possible solutions.

The hypothesis is formulated as an increase of RAE on competitive soccer players during the last years around the world due to the physical advantages that players born in the first months of the year have got in contrast to players born in the last part of the same year.

### 2.2. Procedures and Research Selection

**Figure 1:**
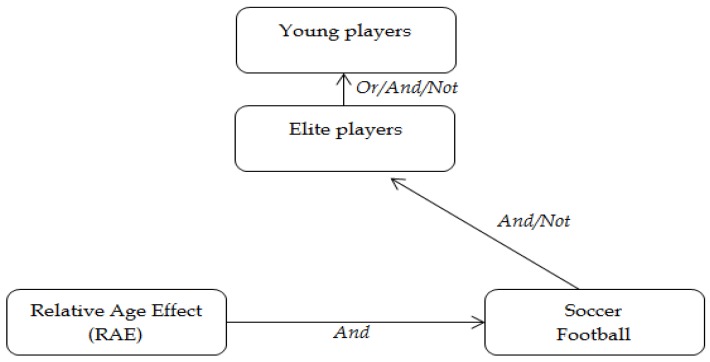
Keywords and Boolean data type used in our search.

The main method used to do a systematic literature search was the use of four reference databases—SPORTDiscus, Medline, EBSCO host and Google Scholar—which include journal papers found in scientific journals related to Sports and Education. The keywords used, as is shown in Figure 1, were ‘Relative Age Effect’, ‘Soccer’, ‘Elite Soccer Players’ and ‘Young Soccer Players’. Additionally, English Boolean data type ‘and’, ‘not’ and ‘or’ were used.

Papers for this review were included according to the following criteria:

(1) Published in English or Spanish.
(2) Published from 2010 to 2016. Articles which define the first concept of RAE were included.
(3) Included information about the RAE among young soccer players and/or elite soccer players.
(4) Included information about the RAE related to maturation, anthropometric characteristics, and physical fitness.
(5) Included information about RAE in several representative countries from all of the five continents.

Review and opinion articles as well as articles focusing on other sports were excluded from this review. Articles which have similar information about the effect in the same country were excluded due to the great amount of papers about RAE around the world.

### 2.3. Bibliographic Selection Process

First, an analysis of the first RAE article was carried out in order to achieve the first definition and its hypothesis [18]. Second, a revision of two review articles [31,32] was carried out to check how RAE increased through several sports as well as how to proceed with the review investigation of this effect. Third, a revision of original papers published by authors that had investigated this effect in soccer during the last six years was performed.

Articles published before 2010 were automatically excluded. On the other hand, articles which compare the RAE between international competitions as well as physical or cognitive characteristics were also analyzed in order to see the relationship between RAE and other factors in young and elite soccer players and to enrich the manuscript. Although the largest number of researchers study RAE and male soccer players, two articles which investigate the effect among female soccer players were included.

According to the criteria selection, 28 manuscripts were chosen. At least two studies were carried out in every year of publication. A table has been drawn up in order to organize the topic as well as to facilitate the comparison between each research paper.

## 3. Results

Table 1 shows the summary of the recent RAE research in elite male and female young soccer players reviewed. Format and design, including the author and the year of publication, the purpose of the study, the participants, the data, the result of the investigation and the relevant conclusions or final discussion are included.

## 4. Discussion

**Figure 2:**
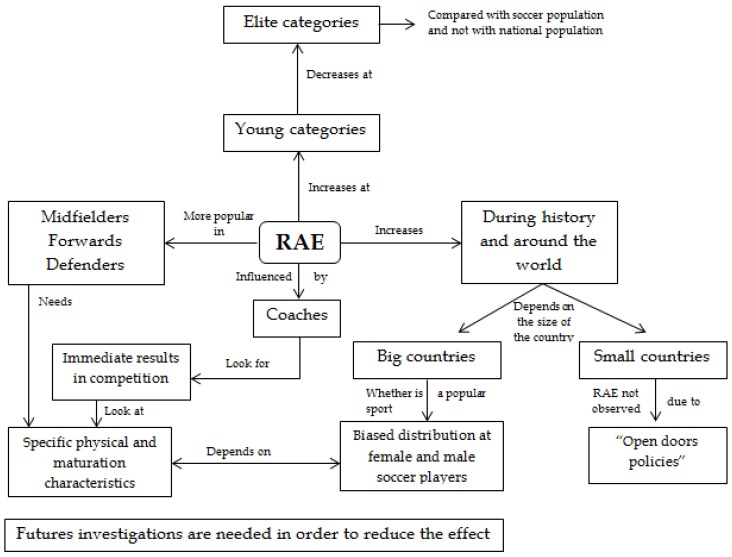
Summary of the topics affected by RAE and its impacts.

The results of the present review confirm the presence of RAE among lower soccer age categories and elite soccer categories around the world. The first date when a RAE study was developed and noted was 1985 [18]. In this line, recent studies reveal that this effect is still present in several sports and has increased in the last two decades [31], especially in popular sport such as soccer [51]. The effect has been examined around the world: America [40,47], Europe [5,7,26,20,29,44] and also in Asia [36,48]. RAE is also investigated alongside several topics (see Figure 2).

### 4.1. RAE among Age Categories

Several studies were observed which compared RAE effects between age categories. RAE seems to decrease at older groups (U-17 and U-18) compared to younger groups [54]. RAE could be due to the minimal differences between players born in the same year in physical and anthropometric variables [51,52]. In this sense, some authors tend to assess the presence of RAE in youth teams using the national population of the country investigated [20,44,47]. However, there are many elite players that come from others inferior teams. So, using global population to observe the RAE in elite clubs could affect the distribution and distort the final results [7]. In these cases, it could be useful to compare the significant participants with the licensed soccer players.

### 4.2. RAE in Female Soccer

Soccer attracts more attention in the male population and competitions are likely to be most popular among male players than female athletes [34]. That is why there is a great amount of studies on RAE in male sports in contrast to studies on RAE in female sports. In this research, two studies which investigate RAE in French and Swiss soccer players were included. Both of them confirm traditional RAE distribution. The effect is also present in the FIFA U-17 Women’s World Cup held in 2008 and 2010 [17]. However, RAE varied among geographical zones. An inverse RAE existed in some parts of Africa, such as Ghana or Nigeria, but not in South Africa [17]. The lack of RAE was also found in Israel [55]. This could be explained by the fact that the number of female players is low [55], as well as the difficulties found to collect data in some African countries [39]. RAE is related to the player position on the pitch [39]. RAE is widely extended in female goalkeepers and defenders due to the physical requirements for these positions [39].

A biased distribution of dropout players was found in the lower categories of age [20]. An over-representation of Q3 and Q4 players, who leave their teams, is influenced by the anthropometric characteristics such as physical growth during the young categories. More investigations are needed in female sport, especially in soccer [34,39].

### 4.3. RAE on the Pitch

RAE in soccer has also been studied depending on player positions. Thus, RAE is more visible at several positions than others. Positions in competition are influenced by specific physical and maturational characteristics. Consequently midfielders, defenders and goalkeepers have been observed as being over-represented by athletes who were born in the first part of the year [26,44]. However, forwards and defenders have been identified as the positions most influenced by RAE in some countries such as Spain [5,45]. In the last years, a change of RAE has been observed in goalkeepers tending to forwards. Children feel more attracted by “more spectacular and amusing” positions, such as midfielders, forwards or defenders. Due to this fact, a great demand of players in these positions has been noted in comparison to others such as goalkeepers [5]. However, defender is the only position in which the RAE effect has been observed permanently. It is explained by the importance of the physical resistance and maturation attributes in this position [5].

### 4.4. Physical Advantages and RAE

In order to observe whether RAE and physical advantages are related to the player selection process, different studies have used several physical tests on soccer players [50,53]. This relationship is suggested to be crucial to determine whether being older means having an advanced biological maturation status (increasing the possibilities to be joined to an important team) [43]. It also helps to explain the great number of players born earlier in the selection year [41]. For example, players who were born in the first part of the year have shown better results in the 15 m test, proving to be faster and more skilled players than athletes who were born in the last quartile of the same year [4]. In addition, older players have revealed an advanced skeletal maturation, were taller, heavier and had long legs. This is also an advantage in competition [46]. However, the use of several physical tests such as the Yo-Yo Intermittent Recovery Test Level 1, does not suggest a significant advantage in terms of soccer-specific endurance among players [41].

### 4.5. RAE around the World. RAE during History

Nations are another topic that has been related with RAE. Every study collects a national or international sample. So, it has been analyzed whether RAE is present everywhere including international competitions. RAE was observed during ten years in male FIFA U17 World Cup teams [56], as well as in the FIFA U-17 Women’s World Cup [17]. It was also observed in the 2012 European Soccer Championship [49], as well as in the FIFA U17 World Cup Emirates 2013 [48].

The size of the country has been revealed as an important factor. In a small country such as Israel, where RAE was not observed, a small number of players interested in a specific sport was noted [36]. Thus, flexible policies (“open doors for everybody”) are suggested in order to recruit players. Amazingly, some African countries that won international competitions had an inversed birthdate distribution (an over-representation of players born in the last month of the year) [48]. This result could be explained by the emphasis on the tactical aspects versus the physical characteristics, although this new hypothesis needs to be assessed. Occidental countries tend to focus on final results, such as long legs or body mass for better technical–tactical aspects, meanwhile other countries seem to value more emotional aspects such as motivation, to the detriment of physical maturation.

In other parts of the world, RAE is increased over the years [40]. In the 2000/01 season, RAE was not observed in Spain and Portugal. However, in the 2010/11 season, only Portugal was observed to avoid RAE [42]. Furthermore, more historical investigations at international competitions are needed in order to observe whether clubs that present high RAE have more probabilities of winning the competition [53].

### 4.6. Practical Application

The findings of this review indicate that the player selection process is unavoidable in a popular sport such as soccer [38]. Coaches usually select players according to an immediate own-need during the season, not taking into account the process of development of the athlete [33]. This could favor the over-representation of the physically advantaged players because younger players, who differ from their older peers, are at risk of leaving their teams [33,35]. Thus, clubs focus on the older players who try to develop as professional players [46]. Although RAE has been identified in the final stage of the competition [38], it does not guarantee success [48]. Indeed, coaches should be properly trained in order to create a homogenous birth-month distribution that prevents dropouts in sport [29]. Coaches hold specific stereotypes about physical size and positive performance attributes among players born in the same year but in different months [15].

In order to make the system more efficient and equal, trainers should be instructed in several important aspects [3,37]. Firstly, they should be sure that the talent promotion is to develop individual potential for elite performance in the future. For instance, tactical skills related to positioning [57]. Secondly, they should be aware of the player perspectives and pay less attention to the momentary level of performance [38]. There are other factors that influence growth and maturation, such as status at birth, household environment, familial correlation, family size, diet and probably others [57].

**Figure 3:**
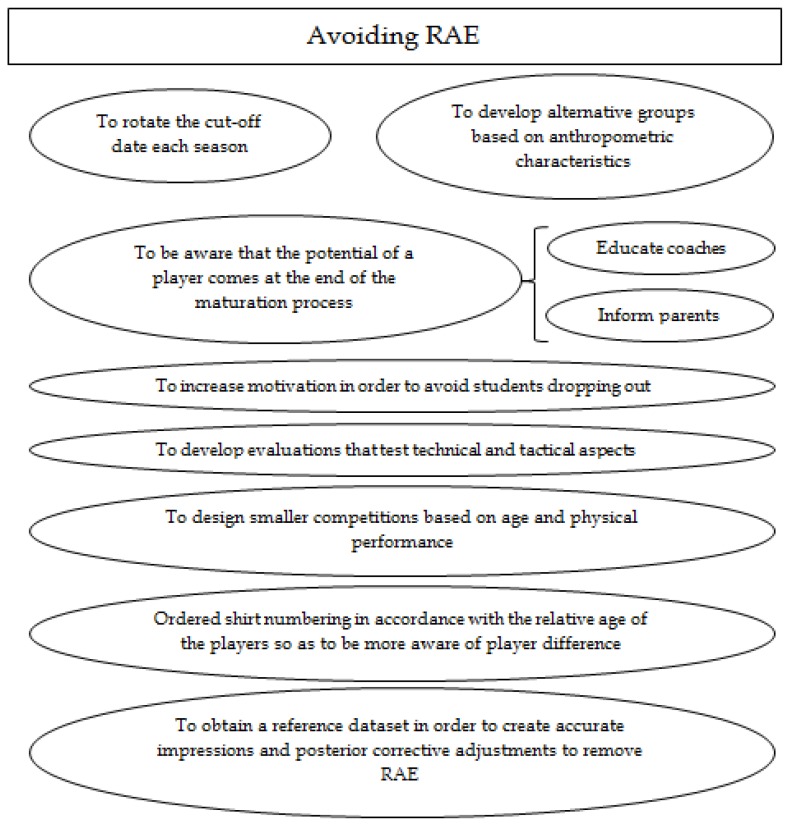
Brief recommendations to avoid RAE in soccer.

RAE represents a bias distribution among young players which seems to decrease among elite players. Solutions should be shown for those professionals who participate in the soccer talent selection as well as in the development process [1,54]. Here, as can be seen in Figure 3, we present some recommendations observed in the manuscripts review to reduce the RAE [20,32]:

A) To alter or rotate the annual cut-off date, which normally is on 31 December, in order to offer different groups of players with a relative age advantage. That could balance the system season after season [6] as well as being fairer [3].
B) To create alternative ways of grouping children for competition based on anthropometric attributes such as height and weight instead of isolated age [1] in order to reduce the maturational effect [49].
C) To develop internal changes in soccer academies as well as improving some rules in the competition. In these cases, clubs could lower the pressure on players taking part in a youth competition in order to avoid dropout players [35], classifying teams according to quarterly periods.
D) To educate coaches and inform parents that the genuine potential of a soccer player comes at the end of the maturation process [20,49].
E) To improve the selection test which guarantees a real evaluation of technical and tactical aspects [28,49] as well as enhancing the playing time for all players, which increases the motivation [52].
F) To design smaller competition groups at young categories as well as group categorized players according to the level of ability which ensures greater opportunity of participation and increases motivation [42,49].
G) To assign numbers on players’ shirts according to their relative age allows coaches to be aware of the personal characteristics of each player. On one hand, this helps coaches to adapt their training to the needs of each player. Conversely, this helps players become aware of skill differences as a result of the relative age differences of their peers [58].
H) To develop corrective adjustments between datasets could help improve the validity of the player’s evaluation and selection process [59].

On the other hand, the bio-banding is the process of grouping players according to their growth and their maturity attributes rather than age [60]. This process could be applicable at competitions, as well as talent identification. Thus, non-invasive methods for estimating maturity status, such as body mass, height, parental height and weight, may allow young talent detection programs to organize players by maturing status rather than chronological age. This could equalize competition and could be a process to avoid RAE [61].

Finally, manuscripts analyzed showed that RAE was observed more often in youth teams in contrast with elite clubs. More research is needed in African countries, where RAE was observed to be lower. More studies about female players have to be studied as well. It is important to improve coaches’ teaching training programs. It could equalize the talent selection system as well as avoiding that high skilled players are named as ‘non-talented’ [28].

## 5. Conclusions

The hypothesis is confirmed: RAE is increasing during the last years in youth elite competition. However, no significant RAE was found in elite soccer teams due to the fact that physical advantages do not exist in older players. It has been observed that RAE depends on several factors, such as physical advantages, the player position on the pitch, the size of the country and the talent detection process. Although recommendations to avoid RAE were proposed, more investigations are needed in order to carry out an effective tool which might reduce RAE. Future research should also focus on the physical characteristics related to sport performance in order to minimize the RAE and balance the quarter distribution during the same year in teams.

## Figures and Tables

**Table 1:** Summary of the investigations related to the RAE.

Authors	Methods			Results	Conclusions
	Aim of the Study	Participants	Data Collect		
Coelho-e-Silva, Figueiredo, Simöes, Seabra, Natal, Vaeyens, Philippaerts, Cumming, and Malina (2010). [33]	Compare the characteristics of regional selected and non-selected players under 14 years of age (U-14) in the first stage of official competition for regional clubs in Portugal as a group as well as by position. Check if the players that were chosen for the regional teams presented a high level of performance on test of function and soccer skill.	A total of 45 regional players and 69 local players in the district of Aveiro and Coimbra.	A mixed-longitudinal study, of the growth, maturation, function and performance around players was carried out. Soccer skill and goal orientation tests as well as training history were included.	Regional players were more experienced in soccer; they obtained better scores in squat jump and running speed. They also were advanced in skeletal maturation, heavier, and had a higher ego orientation. By position, midfielders had a soft higher ego orientation.	Regional coaches could choose players just for immediate competitive needs. Soccer favored early maturing in contrast to late maturing players. Late maturing players at risk of becoming drop-outs and clubs favoring players who are advanced in biological maturation.
Delorme, Boiché, and Raspaud (2010). [7]	Compare the birthdate distribution for the soccer players affiliated to the French Soccer Federation (FSF) and in the French population Test if using all the licensed players versus the national population has an impact on the conclusion of the distribution in elite players.	A total of 1,831,524 French soccer players (including the 351 first division players) during the 2006/07 season.	Birthdates (collected from National Institute of Economical Statistics and Studies) were classified into four quarters (Q) depending on the cut-off date. For elite players, two chi-squared goodness-of-fit test were carried out through StatSoft Inc.	There is an over-representation of players born in Q1 and Q2 and an under-representation of players born in Q3 and Q4. Using the national population to calculate the expected distribution for elite players, significant RAE was showed in the results. However, using the licensed players population biased distribution was not observed.	RAE is present for all age categories in the FSF. The number of dropouts was higher in players born in Q3 and Q4. The great majority of elite players come from a licensed player population. Using the birthdates and distribution of this population and not the national data is better in order to not distort the results obtained.
Delorme, Boiché, and Raspaud (2010). [34]	Observe the presence of RAE depending on female soccer players in French Soccer Federation (FSF) and their age. Investigate whether the effect is related to female dropout from soccer in a diachronic perspective.	A total of 57,892 FSF female soccer players during the 2006/07 season, classifying into six age categories; and 17,285 female players that decided to leave during the 2007/08 season.	Birthdates were obtained from the federation database and were classified into four quarters (Q). In order to see the dropout data, research was based on the birthdates for the whole population of female licensed soccer players, using, in both cases, the weighted mean scores.	Every youth category reflected a classical RAE distribution. However, for the ‘adult’ group, there was an over-representation in Q2 and Q4, and an under-representation in Q3 and Q1. A biased birthdate distribution by quarter of dropout players (during the 2006/07 season) was discovered for the U-10, U-14 and U-17 categories with an under-representation in Q1 and Q2 and an over-representation in Q3 and Q4.	RAE was identified in all categories and seems to decrease with level among females. One of the reasons of these birthdates’ distribution is a higher rate for dropout among players born late in the year as well as a bigger proportion of players born in the first part of the year. It is necessary to continue investigating RAE among female athletes to achieve more data.
Delorme, Boiché, and Raspaud (2010). [35]	Assess whether the RAE is related to dropout behavior observing birthdates’ distribution of French male soccer players who decided to cease their participation during or after the 2006/07 season.	A total of 363,590 French male players during the 2006/07 season that had left their license during the following season.	Players’ birthdates were classified into four quarters. The theoretical distribution is calculated based on the birthdates of licensed players using weighted mean scores.	Dropout rate clearly increases with age in youth categories: From 8.11% of dropout cases in the under 7 years old (U-7) group to 23.49% in adults group. Dropout players in U-9 and U-18 groups born in the last quarter were over-represented.	RAE could be found behind the dropout factor. Children born at the end of the year are dissuaded to engage in sport. However, those who do engage are more prone to dropout from it in a few years.
Gutiérrez-Díaz-Del-Campo, Pastor-Vicedo, González-Víllora, and Contreras-Jordán (2010). [20]	Identify the existence of RAE at youth level in elite and amateur Spanish soccer clubs as well as how RAE has evolved in the last years.	A total of 834 players from 20 clubs in the 2005/06 season. A total of 2,786 players from 20 clubs in the 2008/09 season. A total of 591 amateur players from the 1986/97 Spanish population.	Data collected from Spanish National Institute of Statistics and Royal Spanish Football website. Clubs provided players’ birthdate, position, age group, category of the team and number of years each player has spent in their age group.	The birthdate distribution of players in the groups differed from the Spanish Population as well as between each other. There are big differences between the two elite clubs at the beginning and at the end of the selection year. The distribution of the 2008/09 season players based on the different variables decreases as the number of quarters increases.	RAE in elite soccer is related with the talent identification processes but there are not significant links between other aspects such as the position on the pitch, the category of the team and the number of years they spend in the age group. The presence of RAE can be found in the maturational theory or in the availability of players.
Lidor, Côté, Arnon, Zeev, and Cohen-Maoz (2010). [36]	Examine the RAE and the birthplace effect (how the area influences the players) in elite ball sport players at Division 1, including soccer in Israel, a small country.	A total of 521 ball sport players including basketball (n = 68), handball (n = 161), volleyball (n = 83) and soccer (n = 209).	Five-minute interview for each player, who answer questions about their birthdate and birthplace. The city size was based on Israel census data.	The RAE was not observed in the studied sports. Players’ birthdate distribution was similar in each of the four quartiles. In soccer, there are 28.21% players in the Q1 and 26.84 % players born in the Q3. However, birthplace effect was found here.	Small countries have got a low number of children interested in practicing sports, so coaches should be more flexible. The “open door” policy in clubs is created to recruit the best players for the club.
Wiium, Atle-Lie, Ommundsen, and Eksen (2010). [37]	Examine the presence of RAE within teams belonging to the elite status in the Norwegian Football League.	A total of 217 players between 16 and 38 years old who belonged to 14 Norwegian professional teams during the year 2007.	Birthdate and birth-month were collected from the association of soccer website. Descriptive analysis was run to determine the number and percentage of soccer players born during the different months.	The majority of players were born during the first six months of the year (60%). The month of birth with the highest proportion of players was June. So, players born in the month of June were more likely to be soccer players in the league.	Over-representation of players born in the first part of the year is present in the Norwegian League. RAE is only applicable to a certain degree in the research, so RAE exists partiality in elite soccer. Relationship among birth-month and selection process in elite soccer is not clean.
Auguste, and Lames (2011). [38]	Determine whether German elite under 17 years of age (U-17) soccer teams with significant relative age are more successful versus teams with more equally distributed birthdates.	A total of 911 German U-17 first leagues players from 41 teams for the 2008/09 season.	Birthdates were collected from the homepages of the soccer clubs. In order to identify the RAE, a Kolmogorov–Smirnov test was carried out. Median of birthdates was calculated as a measure of the effect size of RAE. Finally, indicators of teams’ success were examined as the variable rank as well as goals and points achieved.	There is a significant tendency to prefer earlier born players in the three leagues. There is a medium relative age effect in the average of all clubs. The team with the smallest effect ended 13th in the league. Teams with a high RAE achieve more points, score more goals (but it is not significant) and concede fewer goals. The team with the largest RAE is more likely to finish 5.5 ranks higher than teams with small RAE.	RAE is significant among elite male U-17 soccer players. There is a relationship between this effect and final rankings for the teams. So, to be successful in the high leagues, coaches prefer older players who are more physically mature and show better performance. The immediate success of the teams is more important than promoting the most promising players into the elite league at adult age.
Lesma, Pérez-González, and Salinero (2011). [26]	Assess whether RAE is present in the Spanish Football League.	A total of 481 elite soccer players of the Liga BBVA (which is every player of the league during the 2009/10 season).	Birthdates and position on the pitch were collected from the 2009/10 Marca League Guide. All the information was checked with the website of the teams. Analyses were carried out with statistical package SPSS v.18.	There is a RAE of 61.12/38.88 (percentage of players born in the first quarter of the year/percentage of the rest of the players). RAE is also shown in the international players of the league [RAE of 63.53/36.47]. RAE is more significant in defenders, midfielders and goalkeepers.	RAE is significant in the league. This sport is very popular and generates a lot of interest. Clubs select players who were born in the first part of the year because they show physical and maturity advantages. Working in the way to improve the talent selection could be the key to avoiding RAE.
Romann, and Fuchslocher (2011). [39]	Examine the prevalence and the size of RAE at the different age and performance levels in Switzerland. Observe the relationship between RAE and the playing position.	A total of 6229 Swiss female soccer players grouped in different levels of selection in Switzerland	The birth-month of each player was collected to elaborate the birth quarter. (The year was divided into four quarters).	The effect was observed among young players (from 10 to 14 years). The peak of RAE was found in the U-10 and U-11 teams (66.6% of the female players were born in the first part of the year).	RAE is present in defenders and goalkeepers. However, RAE is not present in the highest selection levels. RAE does not influence the talent identification process at elite teams.
Teoldo, Albuquerque, and Garganta (2012). [40]	Evaluate the existence of RAE in Brazilian football players throughout history.	A total of 202,951 players from the Brazilian Football Confederation (CBF) born from 1921 to 1996. Every participant was registered as a professional football player until 2011.	Participants’ birthdates were collected from the CBF database. In order to examine RAE, birthdates distribution was categorized into four quarters. The categories of birth-decade were used as well to group players and identify changes in trends during the history.	RAE is present in Brazilian soccer players. The effect is not significant until 1960, when RAE has progressively increased over the years. In 1960, physical preparation was adopted by coaches in soccer clubs around Brazil. The standardized residual per quarter shows differences between Q1 and Q2 in contrast to Q3 and Q4 during 60th, 70th, 80th and 90th decade.	The actual selection process in youth players can fail in order to provide opportunities for players born at the end of the year. An historical analysis of the RAE should be performed in European countries (where soccer is well-consolidated) as well as in African countries (where soccer is still not consolidated).
Deprez, Vaeyens, Coutts, Lenoir, and Philippaerts (2012). [41]	Observe the distribution of birthdates in elite Flemish youth soccer players. Assess the influence of the RAE and an estimation of biological maturity on anthropometric characteristics and performance in Yo-Yo Intermittent Recovery Test Level One (Yo-Yo IR1) among the four birth quarters (Q) of the selection year.	A total of 606 elite youth soccer players from two clubs from the Belgian first division born between 1988 and 2001.	Players were classified into five age categories. Each category was divided into four birth-quarter and two semesters. Cut-off date on 1 January. Anthropometric measures, maturity status and Yo-Yo IR1 were carried out in this mixed-longitudinal study.	A total of 67.2% of the total sample was born in the first semester of the selection year. With the MANCOVA analysis, there is no significant effect for birth quarter within all age categories, although there was a significant effect of chronological age on the Yo-Yo IR1 performance in all categories except in U-10, U-11 and U-12, U-13.	There was an over-representation of players born in the first birth quarters that were taller and heavier than players born in the last quarters. APHV did not influence the Yo-Yo IR1 performance. There are not differences in intermittent endurance performance among early and late born players.
Helsen, Baker, Michiels, Schorer, Van-Winckel, and Williams (2012). [42]	Observe the birthdate distribution in the important soccer competitions in Europe over a ten-year period involving the 2000/01 and the 2010/11 seasons.	Every professional player in the 2000/01 and 2010/11 seasons was examined (n = 9336).	Birthdates were collected from the website of the clubs. Cut-off date was changed depending of the season. The distribution of the 2000/01 season was compared with the other season distribution using a Chi-square test.	There was RAE in both seasons. If the percentage of home country players was considered, RAE is shown to be stronger in the 2010/11 season than in the other. In both seasons, most of the birthdates were asymmetrical. Comparing the birth-quartile distribution of the seasons, RAE was increasing in several countries.	In the 2000/01 season, just Spain and Portugal did not show RAE. In the other season, just Portugal keeps away from RAE. Prevalence of the REA does not seem to have decreased over the last ten years. Rotating the cut-off date could be a solution to reduce RAE in talent detection.
Deprez, Coutts, Fransen, Deconinck, Lenoir, Vaeyens, and Philippaerts (2013). [43]	Investigate the influence of the RAE in the birth quarter on anthropometry, biological maturity and anaerobic parameters.	A total of 374 elite Belgian youth soccer players.	Players were divided into three groups; each group was subdivided into four birth quarters. APHV estimated, height, weight, SBJ, CMJ, 5 and 30 meter sprint were collected.	A total of 42.3% of the participants were born in the first part of the year in contrast with 13.7% players born in the last part of the same year.	Youngest players can balance the RAE if they enter into puberty earlier. Estimating differences among players because of the large discrepancies between statistical and practical significance should be taken into account carefully.
Gil, Badiola, Bidaurrazaga-Letona, Zabala-Lili, Gravina, Santos-Concejero, Lekue, and Granados (2013). [4]	Assess whether RAE is present in young soccer players. Evaluated whether players born at the beginning of the year would have some physical advantages as well as having better performance than the others players.	A total of 88 voluntary young soccer players from Bizkaia soccer clubs who were born in 2001.	Several physical tests were carried out including anthropometric measurements; maturity; velocity, agility, jump and a hand dynamometry test and a Yo-Yo Intermittent Recovery Test. Each test was performed three times (except the last one) and the best marks were used.	A total of 65.91% were born in the first half of the year. There are significant differences among the four quarters of birth. The older players were taller and had longer legs, performed better in velocity and agility tests, in the handgrip test as well as the Yo-Yo Intermittent Recovery Test.	RAE was confirmed. Reasons for RAE are not clean and larger studies are needed in order to confirm the relationship between physical performance, body size and RAE. Being tall and having longer legs is a physical advantage for soccer players. Competition to obtain a place in a team could be one cause of RAE, which depends on the population.
Romann, and Fuchslocher (2013). [44]	Determine the existence, the prevalence as well as the size of RAE (within the different levels of Swiss junior soccer) and their link with the playing position.	A total of 50,581 Jugend und Sport (J + S) players aged 10 to 20 (level 1), 2880 talent development players aged 10 to 20 (level 2) and 630 national junior players aged 14 to 20 (level 3).	In order to define the birth quarter, the birth-month of each player was collected from the Swiss Federal Office of Sport. Birthdate distributions were calculated for each quarter. Distribution of J + S was used as a basis in order to evaluate RAE. Swiss population was used to compare the data.	There are no significant differences between RAE compared with the entire Swiss population and all J + S players. The effect was huge for (under 15 years old) U-15, U-16, U-17, and U-18; medium for U-19 and U-21; and small for U-20. There are also significant differences among defenders, strikers, midfielders and goalkeepers compared to the J+S distribution. A total of 79% of defenders were born in the first half of the year.	RAE influences the selection process of elite Swiss soccer in the second and the third level. There is also a relationship between this effect and playing position in this country. A significantly higher effect was observed for defenders compared to midfielders, goalkeepers and strikers. Those who were born early in the year are five times more likely to be selected to perform in the U-15 team.
Salinero, Pérez, Burillo, and Lesma (2013). [45]	Confirm the presence of the RAE in professional soccer in European competitions as well as to evaluate the influence of the playing position on this effect.	A total of 2763 players from five European leagues including, United Kingdom, Italy, Germany, France and Spain.	The playing position as well as the date of birth of every player was collected from the websites of the different teams. Analyses were carried out with statistical package SPSS v.18 and frequencies were obtained for quarters.	The first quarter was over represented in contrast to the other three. RAE is confirmed in elite soccer players in Italy, France and Spain. The positions which present the highest RAE level were midfielders and defenders. In the Premier league RAE is not significant in midfielders.	The analysis by position demonstrated that this aspect played an important role in the RAE, and is evident at the international level. The characteristics of the position in the competition could influence the talent selection as well as the RAE.
Fragoso, Massuca, and Ferreira (2014). [46]	Ascertain the relationship between birthdate distribution, biological maturity as well as physical fitness in Portuguese under 15 years old (U-15) soccer players.	A total of 133 U.15 soccer players from a Portuguese top elite soccer academy.	The collection period lasted from the 2002/03 to 2009/10 season. Participants’ birthdates were classified by birth quarters and by semesters. Biological maturity, anthropometric variables and fitness profiling tests were carried out.	Differences are clean between skeletal and decimal age compared with birth quarters and with semester. There is a significant stature differences between all of the birth quarters. There are also differences between birth quarters and 10-m and 30-m sprint time tests as well as squat jump test results. If the effect of the skeletal age was removed, the differences observed disappeared.	Players born in the first part of the year show fitness advantages. There is a clear relationship between birth distribution and biological maturity. Besides, players born at the beginning of the year tend to mature early and are more frequently selected. However, focusing on present physical attributes could be a risk of talent loss.
Massa, Caldas-Costa, Moreira, Rogérin-Thiengo, Rodrigues-de-Lima, Quispe-Marquez, and Saldanha-Aoki (2014). [47]	Compare the birthdate distribution of youth players from 10 to 20 years old of a high-level Brazilian soccer club with the same age population in São Paulo from 1991 to 2001.	A total of 341 youth athletes from the São Paulo Football Club who played in 2011.	Players were divided into nine categories (from U-10 to U-20) to carry out a cross-sectional study, as well as being classified into four quarters depending on the birthdate. Births in São Paulo were collected to compare the players’ birthdate distribution.	Less than 10% players were born in the fourth quarter. Although there are not significant differences in the birthdate distribution of the general population, there is high incidence of RAE in youth soccer players, especially in the U-10 and U-17 categories. Uniform birthdates distribution was observed for the U-20 category.	RAE is a global phenomenon that not only occurs in Europe. However, this effect is a highly-debated topic. There are various issues related to this phenomenon that are not clean. That is the reason why national studies are needed to identify the strongest relationship between factors and the occurrence of RAE.
Mulazimoglu (2014). [29]	Observe the influence of RAE on the Turkish Super League (TSL) as well as all competitor categories of youth teams in Turkey.	A total of 2939 professionals and young soccer players in 18 clubs of the TSL during the 2013/14 season.	Data were collected from the website of the Turkish Football Federation. Chi-squared statistic test was used to compare differences between the observed and expected birthdate.	The categories have got significant differences between the birthdate in the first months of the year [1315 players were born in January]. They present a linear decrease during the whole year except professional teams. U-17, U-15 and U-14 present the highest percentages.	RAE is present on the top clubs in Turkey. This effect is more common among youth competitions. However, it decreases in professional levels. Trainers should be educated in order to avoid dropouts and to provide equal opportunities.
Andrade-Souza, Moniz, and Teoldo (2015). [48]	Assess whether the birthdate is a decisive factor among successful players who are selected to participate in the FIFA under 17 years of age (U-17) World Cup Emirates 2013.	A total of 503 U-17 FIFA World Cup Emirates players (from 24 countries) during the 2013 competition.	Birthdates of the players were collected from the FIFA website and were classified into four quarters (Q) and into stages according to the competition. The cut-off date was on 1 January. Birthdate distributions of athletes who effectively play were included.	There was an over-representation of players born in the first part of the year in the group stage, round of 16, quarter-final and semi-final. There were no significant differences among Q1 players and Q4 players at the final. Nigeria was the U-17 World Cup champion. It showed an inverse birth-month distribution with 26.1% of the players born in Q4 in contrast with the 4.4% born in Q1.	RAE was observed up to the semi-final stage. Significant differences in maturity, psychological or physical fitness among players in a national team remind that RAE is clearly present in national competitions. However, in African regions we could observe a reverse effect (the champion in 2013 was Nigeria). Other issues should be taken into account to become champion.
González-Víllora, Pastor-Vicedo, and Cordente (2015). [49]	Examine the birthdates of 2012 European Soccer Championship players at a senior level and in under 21 years old (U-21), U-19 as well as U-17 from the previous European Soccer Championship in order to identify the existence of RAE in UEFA (Union des Associations Européennes de Football).	A total of 841 players from 16 teams participated in the Eurocup of 2012. A total of 184 players from U-21 at 2011. A total of 144 players form U-19 at 2012. A total of 145 players form U-17 in 2012.	Data collected comes from the website of the UEFA. That included the player´s name, day, month and year of birth as well as the country the player comes from, the category and the playing position. Only the teams that had participated in the final stages were considered.	RAE is not detected in the professional category meanwhile RAE is observed in the U-19, U-21 and U-17. Although it is more significant in the final match of U-19, RAE is much higher in the semifinal of the U-17 category compared to the U-21 category. RAE increases among teams which participated in the semifinals as well as teams that won international tournaments.	RAE was not evident in the professional category but it was in the three lower categories. RAE is observed in professional soccer and seems to increase in the teams which reach the final stages. The hypothesis to explain RAE should be that it is related to athletic performance, to physical cardiorespiratory condition, to maturation or to experience.
Lovell, Towlson, Parkin, Portas, Vaeyens, and Cobley (2015). [50]	Investigate the magnitude of the RAE in each annual age-group. Confirm the relationship among relative age and maturation status, anthropometric performance as well as physical performance across youth English soccer players’ pathway.	A total of 1212 players between 9 and 18 years old who come from 17 English soccer clubs.	Players were classified into age-group categories birth quartiles (Q). Players must complete a standardized battery assessments to collect anthropometric attributes, maturation status as well as physical fitness capacities.	A total of 36%–56% of players born in Q1 in contrast to 7%–16% born in Q4. An over-representation of Q1 players was identified in U-9, U-13 and U-16. Depending on the age category, Q1 presents higher body mass and structure versus Q4. There are differences in physical sites according to age category as well as a number of anaerobic performance advantages in Q1.	RAE is confirmed in the English lower-league professional clubs. The RAE upon anthropometric or performance characteristics was absent in U-16 and U-18 groups. RAE is significantly high at U9 and U-10 but it is softly decreasing at U-11, U-12, U-13 and U-14. RAE is very high at U-15 and U-14. However, it is reduced in the last groups.
Mikulič, Gregora, Benkovský, and Peráček (2015). [51]	Verify whether RAE exists in the Slovakia national football teams from the under 16 age category (U-16) to the A-senior national football team.	A total of 79 Slovak U-16 players, 47 U-17 players, 58 U-18 players, 71 U-19 players, 52 U-21 players and 302 A-senior players.	Data were collected from the Slovak Association. Statistical methods, analysis, inductive and deductive approaches were applied in the data processing and evaluation.	RAE is present in all youth groups, except at U-19, U-21 and senior teams. Football coaches are influenced by the relative age effect. However, maximum maturity in sports occurs at the age of 21.	RAE decreases with the increase of age. Anthropometric factors did not influence a team. Physical and maturation parameters are balanced in the adult age among players born in the same year.
Prieto-Ayuso, Pastor-Vicedo, Serra-Olivares, and González-Víllora (2015). [5]	Check the existence of RAE in Spanish Soccer in session 2013/14 keeping in mind the birthdate of the players, the teams, the nationality as well as the position on the field.	Every elite soccer player in the 20 teams of the first division in the First Division of Spanish Soccer League. (n = 474).	Birthdate, team, position on the field and nationality were collected from the 2013/14 Marca League Guide.	There were significant differences in the birthdates in several popular Spanish teams with an over-representation of players born in the first part of the year. RAE is observed in determined positions on the pitch. There was also an over-representation of international players born in the first six months of the year.	RAE is present in the BBVA League. Analyzing others seasons, there is a soft increase concerning RAE. The effect is popular in forwards and defenders due to the popularity of sport. However, it is vital to continue future investigations about this effect. RAE is very present in forwards and defenders.
Hill, and Sotiriadou (2016). [28]	Check whether coach awareness helps to eliminate RAE during talent selection as well as to evaluate whether the decision-making (DM) process influences RAE.	A total of 263 young male football players (11–15 years) in Australia and four coaches.	Divided into three visual and verbal phases for the coaches. SPSS v.21 was used to select groups. Semi-structured interviews with qualitative data were used in the process.	RAE continues to emerge among players selected as talented even though coaches were consciously aware of its effect. Players in the birth-month group January–April had a 1.33–2.33 chance of being selected as talented depending upon the age group of the player.	Increasing awareness of RAE did not result in any significant changes in DM. Isolated coach education is not sufficient to introduce a change in their DM and talent selection. There should be more recommendations on organization.
Arve-Sæther (2016). [52]	Observe the presence of RAE among U-17 and U-20 players, in a four-year period in the Norwegian premier league Tippeligaen as well as to assess the connection between RAE and the amount of playing time.	A total of 315 male soccer players born in 1990/96 given playing time in the 2009/12 period.	Information was collected from a website. Players’ birthdates were categorized into quarters. A second analysis focuses on the age categories and the birthdate.	A total of 68% of the players were born in the first quartiles of the year. The tendency also increases from 2009 to 2011. There is a clear connection between the birth-month in the first part of the year and the highest amount of playing time.	There are few studies of the RAE among youth players in top-level clubs. RAE is a relevant factor in the selection process of elite youth soccer players in Norway. Players are selected by their birth-month.
Skorski, Skorski, Faude, Hammes, and Meyer (2016). [53]	Investigate whether anthropometric profiles and fitness measures vary according to birth-date in the German national youth soccer teams. Analyze whether there is a difference in the chance of becoming a professional player.	A total of 554 players (divided into six age groups). A total of 832 data sets from 495 individual soccer players (born from 1987 to 1995) were included	Players were divided into six age groups and performed some tests to determine the anaerobic threshold, the countermovement jump and the 30-m sprint. There were tests within the team and the best one was included.	More players were born in the birth-quarter 1 (BQ1) than in all other BQs. Players born in BQ4 were more likely to become professional than those born in BQ1. No significant difference at BQs is observed in any anthropometric or performance characteristics.	RAE exists in elite German youth soccer. RAE does not explain the advantage in anthropometric or performance-related parameters. Younger players selected into national teams have a greater chance of becoming professionals in their career.

## Abbreviations

The following abbreviations were used in this manuscript:

Age at peak height velocity	(APHV)
Birth quarters	(Q)
Counter movement jump	(CMJ)
Fédération Internationale de Football Association	(FIFA)
Multivariate analysis of covariance	(MANCOVA)
Standing broad jump	(SBJ)
Under n° years old	(U-n°)
